# Evolution of molecular diagnostic strategies for 21-hydroxylase deficiency: from classical methods to advanced genomic techniques

**DOI:** 10.3389/fendo.2026.1770274

**Published:** 2026-04-02

**Authors:** Mirela Costa de Miranda, Ana Carolina Maués de Oliveira, Gabriel Junqueira Soares, Júlio Américo Pereira Batatinha, Ana Claudia Latronico, Berenice Bilharinho Mendonca, Tania A. S. S. Bachega

**Affiliations:** Laboratório de Hormônios e Genética Molecular - LIM/42, Unidade de Desenvolvimento, Disciplina de Endocrinologia e Metabologia, Hospital das Clínicas, Faculdade de Medicina da Universidade de São Paulo, São Paulo, Brazil

**Keywords:** 21-OHD genotype, congenital adrenal hyperplasia, *CYP21A2* molecular diagnosis, long-read sequencing, next-generation sequencing

## Abstract

Congenital adrenal hyperplasia (CAH) due to 21-hydroxylase deficiency is one of the most technically challenging monogenic conditions for molecular diagnosis, owing to the complex genomic architecture of the *CYP21A2* locus and the extensive homology between the functional gene and its pseudogene. Over the past decades, diagnostic approaches have evolved from locus-specific assays, such as allele-specific polymerase chain reaction, to the combined use of Sanger sequencing and multiplex ligation-dependent probe amplification (MLPA), which together constituted the historical gold-standard approach strategy capable of identifying pathogenic variants, deletions and chimeric alleles. Although short-read next-generation sequencing expanded variant detection and enabled simultaneous analysis of multiple adrenal genes, its performance remains limited within the *RCCX* region due to misalignment artefacts and inability to resolve complex structural rearrangements. Recently, long-read sequencing (LRS) has emerged as a single-platform technology capable of resolving the *CYP21A2–CYP21A1P* module, accurately detecting all variant classes and directly determining cis/trans phase. Comparative studies demonstrated complete concordance with standard methods while revealing additional rearrangements previously undetectable, positioning LRS as a future reference method for high-resolution genotyping. These advances extend beyond diagnostic refinement to impact population-level strategies, where integration of molecular testing into newborn screening algorithms reduces false positives, accelerates referral pathways, and enhances salt-wasting risk prediction. In this review, we summarized the historical development, technical limitations, and clinical implications of current and emerging molecular approaches for CAH diagnosis. We highlight how LRS and integrative analytical tools are reshaping clinical practice, refining genetic counselling, and guiding personalized therapeutic strategies. Collectively, these innovations represent a decisive step toward precision Endocrinology.

## Introduction

Congenital adrenal hyperplasia (CAH) encompasses a group of autosomal recessive disorders affecting adrenal steroidogenesis, resulting in impaired synthesis of cortisol and aldosterone. Approximately 90–95% of all CAH cases are caused by 21-hydroxylase deficiency (21-OHD), due to pathogenic variants in the *CYP21A2* gene (1). Loss of 21-hydroxylase activity impairs the conversion of progesterone and 17-hydroxyprogesterone (17-OHP) into downstream metabolites, leading to cortisol and aldosterone deficiency, excessive adrenocorticotropic hormone (ACTH) stimulation, adrenal hyperplasia, accumulation of androgen precursors and excess of androgen production, which explains the virilization characteristic of the disorder (1).

The first detailed description of CAH dates back to 1865, documenting the life and post-mortem findings of Giuseppe Marzo, a phenotypic male who possessed internal female reproductive organs and markedly enlarged adrenal glands who died at the age of 44 during an apparent Addisonian crisis (2). In 1950, Wilkins and colleagues (3) introduced cortisone therapy for classical forms, revolutionizing the disease’s natural course. Years later, Bongiovanni and Root (4) identified 21-OHD as the biochemical cause of CAH, linking the disorder to a specific enzymatic defect in the steroidogenic pathway.

The 21-OHD exhibits a broad phenotypic spectrum, determined by the degree of residual enzymatic activity. Affecting 1:13,000 to 1:15,000 live births, the classical forms (salt-wasting-SW and simple-virilizing-SV) are the most severe, presenting with prenatal androgen excess leading to virilized external genitalia in 46, XX newborns (1). The SW form, accounting for 75-90% of the classical cases, additionally causes life-threatening aldosterone deficiency, resulting in salt-wasting crises, representing the most severe phenotype of 21-OHD. Without treatment, both classical forms may cause postnatal progressive virilization, advanced bone age, and early pubarche. In contrast, the nonclassical (NC) form is milder, with normal genitalia at birth and later-onset hyperandrogenic features (5).

Clinical assessment and hormonal profiling are essential for diagnosing 21-OHD, with serum 17-OHP levels serving as the key biochemical marker of the disease. Due to the risk of lethal salt-wasting crisis, 17-OHP measurement is included in many standard newborn screening programs worldwide (6). In classic forms, 17-OHP levels are markedly elevated, particularly in SW cases; however, its concentration alone may be insufficient to distinguish between the SW and SV subtypes (7). In NC forms, 17-OHP may be only mildly elevated, often requiring ACTH stimulation for confirmation (8). Given biochemical limitations in borderline results, and atypical presentations, molecular testing plays an increasingly important role in confirming the diagnosis, predicting disease severity and guiding genetic counseling.

It was not until the 1980s, three decades after the introduction of cortisone therapy, that the cloning and characterization of the *CYP21A2* gene marked a turning point in the understanding of 21-OHD. This milestone revealed the presence of a highly homologous pseudogene, *CYP21A1P*, located within the complex *RCCX* module, an insight that helped explain the high frequency of recombination events underlying the disorder (9, 10). These early molecular discoveries laid the groundwork for subsequent advances in genetic analysis, which would later refine diagnostic accuracy and deepen our understanding of disease heterogeneity.

Over decades, molecular diagnosis evolved from labor-intensive, low-resolution techniques to increasingly comprehensive approaches. While Sanger sequencing combined with multiplex ligation-dependent probe amplification (MLPA) constituted the classical standard, limitations remained in resolving complex gene rearrangements and allelic phase. Short-read Next-Generation Sequencing (NGS) expanded variant detection but continued to face interference from the pseudogene. Most recently, long-read sequencing has emerged as a transformative single-assay approach, providing near-complete *CYP21A2* characterization, including haplotyping, structural variant detection, and cis/trans phasing (11, 12).

As genetic insights increasingly shape diagnostic accuracy and therapeutic decision-making, a comprehensive overview of the molecular foundations of the disorder becomes essential. This review synthesizes current knowledge on the genetic architecture of the *CYP21 locus* and the evolution of its molecular diagnostic methodologies, highlighting future perspectives toward faster, more accurate, and clinically applicable diagnosis of CAH. Variant nomenclature follows Human Genome Variation Society (HGVS) recommendations and is based on the *CYP21A2* reference sequence NM_000500.9 (protein: NP_000491.4). Legacy names are provided where relevant to align with earlier literature.

## Genetic background of the *CYP21A2* gene and CAH due 21-OH deficiency

**Figure 1:**
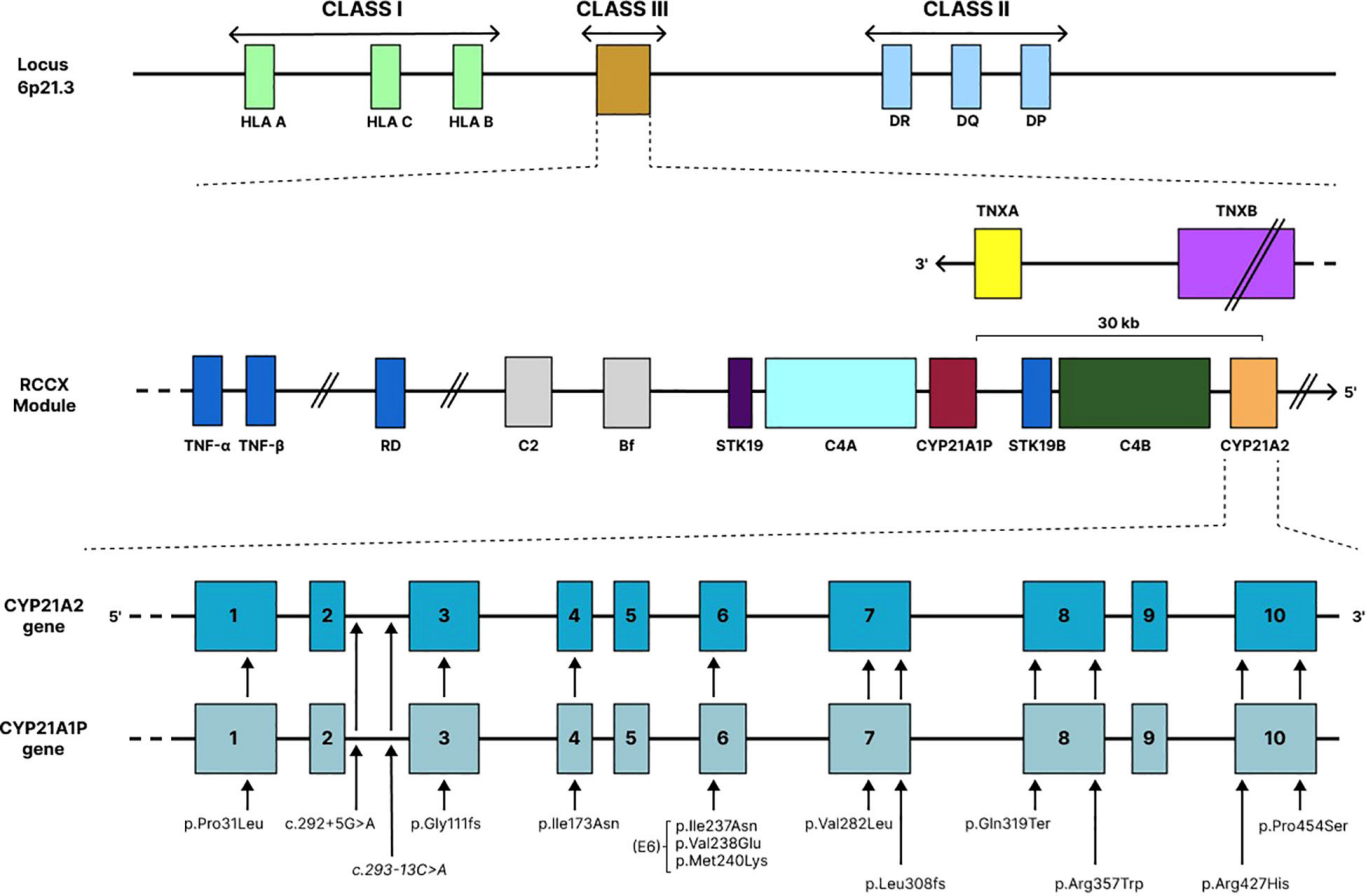
Locus RCCX: Schematic representation of the RCCX locus within the MHC region on chromosome 6p21.3, showing the modular arrangement RP–C4–CYP21A1P/CYP21A2–TNX. The functional CYP21A2 gene resides adjacent to its highly homologous pseudogene CYP21A1P, while flanking TNXA/TNXB genes predispose this region to non-allelic homologous recombination, leading to gene conversions, deletions, and chimeric alleles associated with congenital adrenal hyperplasia. Major CYP21A2 pathogenic variants derived from the CYP21A1P pseudogene across the exonic structure of CYP21A2: Schematic representation of the ten exons of the CYP21A2 gene indicating the location of the most frequent pseudogene-derived pathogenic variants associated with 21-hydroxylase deficiency, including p.(Pro31Leu), p.(Gly111fs), c.293-13C>G, p.(Ile173Asn), exon 6 cluster (p.[Ile237Asn], p.[Val238Glu], p.[Met240Lys]), p.(Val282Leu), p.(Leu308fs), p.(Gln319Ter), p.(Arg357Trp), and p.(Pro454Ser). These variants arise predominantly through microconversion events from CYP21A1P and represent the majority of disease-causing alleles worldwide. Adapted from Concolino et al (16). This schematic representation is not drawn to scale. Exon sizes, intergenic distances, and relative gene lengths are depicted for illustrative purposes only. Arrows indicate regions of gene conversion or recombination events within the RCCX locus and do not represent physical distances.

The *CYP21A2* gene is located within the *RCCX* module on chromosome 6p21.3, in class III region of the major histocompatibility complex (MHC). This region spans approximately 30 kb and is characterized by a complex arrangement of tandemly repeated genes, namely *C4A/C4B*, *CYP21A1P/CYP21A2*, *TNXA/TNXB (Tenascin X)*, and *STK19/STK19B (Serine/Threonin Kinase –* formerly *RP1/RP2)* (13–15). The functional gene *CYP21A2*, located adjacent to *C4B*, extends over approximately 3.4 kb and encodes a cytochrome P450 enzyme comprising 495 amino acids, whereas *CYP21A1P*, located adjacent to *C4A*, is a pseudogene containing multiple inactivating pathogenic variants that preclude proper protein expression (9, 10) (**Figure 1**). Both genes contain 10 exons and share remarkably high sequence identity, approximately 98% identity in exons and 96% in introns (9, 10).

The *CYP21A1P* pseudogene is inactive because it harbors numerous deleterious variants across its promoter and coding regions, including seven missense substitutions, an exon 3 frameshift, the intron 2 splice change c.293-13C>G, and truncating or frameshift variants in exons 7 and 8. The three exon 6 substitutions [p.(Ile237Asn), p.(Val238Glu), p.(Met240Lys)] constitute the classic “E6 cluster,” while promoter single nucleotide variations (SNVs) further reduce transcription to ~20% of normal. Together, these genetic changes abolish enzymatic activity and render *CYP21A1P* the primary donor sequence in gene conversion events affecting *CYP21A2* (16). The abovementioned high degree of homology and tandem configuration make this region particularly prone to meiotic misalignment, which fosters recurrent recombination through unequal crossover and gene conversion. These processes constitute the predominant mechanisms underlying pathogenic variants in 21-OHD (16–19).

Overall, 90–95% of pathogenic *CYP21A2* variants arise from intergenic recombination between the active gene and its pseudogene within the *RCCX* module. As a result, a limited set of pathogenic variants with well-established phenotypic consequences is consistently observed across all populations (**Tables 1**, **2**). Among these, microconversion events, in which one or more deleterious pseudogene-derived variants are transferred into *CYP21A2* during meiosis, represent 70–75% of all cases (5, 16, 19, 20). The remaining 20–25% are attributable to unequal meiotic crossovers, giving rise to extensive genomic rearrangements, including large deletions, duplications, and the formation of chimeric *CYP21A1P/CYP21A2* genes. In contrast, only 5-10% of small pathogenic variants in *CYP21A2* occur independently of gene conversion events; some of these have founder effects, providing valuable insights into population history and migration patterns (5, 16, 19–22).

**Table 1:** Description, genomic location, and residual enzymatic activity of the most frequent CYP21A2 pathogenic variants, including large gene rearrangements (whole-gene deletions and gene conversions) and recurrent point mutations, reported in different populations.

Pathogenic variant	Location	Enzymatic activity (%)*	Associated phenotype	Group	Presence in pseudogene
Whole-Gene deletion	Entire gene	0	SW	Null	—
Large *CYP21A2* rearrangements**	Exons 3-8	0	SW	Null	—
p.(Pro31Leu)	Exon 1	30-60	NC	C	Yes
c.293-13C>G	Intron 2	<2	SW/SV	A	Yes
8-bp deletion***	Exon 3	0	SW	Null	Yes
p.(Ile173Asn)	Exon 4	3-7	SV	B	Yes
E6 Cluster****	Exon 6	0	SW	Null	Yes
p.(Val282Leu)	Exon 7	18	NC	C	Yes
p.(Leu308Phefs*21)	Exon 7	0	SW	Null	Yes
p.(Gln319Ter)	Exon 8	0	SW	Null	Yes
p.(Arg357Trp)	Exon 8	2	SW/SV	A	Yes
p.(Pro454Ser)	Exon 10	20-66	NC	C	Yes

*The effect of each variant on enzymatic activity is listed as percent of wild-type activity when mutant cDNA is transfected into cultured cells and assayed in intact cells. Enzymatic activity values are reported as ranges, reflecting data derived from different experimental and cohorts’ studies. Because these estimates originate from heterogeneous *in vitro* expression models and assay conditions, direct quantitative comparisons across variants should be interpreted with caution. **CYP21A2 large gene conversion refer to large structural rearrangements involving transfer of pseudogene-derived sequences extending from somewhere between exons 3 and 8 of *CYP21A1P* to the corresponding point in *CYP21A2*, yielding a non-functional gene in which the 5’-end corresponds to *CYP21A1P* and the 3’-end corresponds to *CYP21A2*, leading to complete loss of 21-hydroxylase activity, and are therefore classified as null alleles. Those are called gene chimeras. Whereas complete deletions generally affect the entire CYP21A2 gene ***8-bp deletion = c.332_339del, ****Exon 6 cluster = p.[Ile237Asn; Val238Glu; Met240Lys] Adapted from Speiser 1992, White 1994 and Concolino 2025 (16, 48, 50).

**Table 2:** Frequency of CYP21A2 mutations across countries.

Country reference	Aleles (n)*	Del/Conv (%)	p.(Pro31Leu) (%)	c.293-13C>G (%)	c.332_339del (%)	p.(Ile173Asn) (%)	E6 cluster** (%)	p.(Val282Leu) (%)	p.L308fs (%)	p.(Gln319Ter) (%)	p.(Arg357Trp) (%)	p.(Pro454Ser) (%)	Variants derived from the pseudogene (%)
Sweden (31)	186	29.8	8.5	30.0	2.1	20.3	0.0	7.0	0.5	3.2	3.6	NR	100
Mexico (32)	94	1.0	NR	47.9	1.0	11.7	NR	8.5	1.6	4.2	2.4	NR	94.5
Chile (71, 72)	164	19.5	1.6	15.8	1.6	7.3	1.1	2.4	0.0	7.9	4.5	2.1	64.4
Spain (94)	354	19.2	2.6	17.5	3.3	3.7	1.0	33.9	1.4	9.3	9.7	0.5	93.9
Central Europe (30)	864	30.6	NR	31.2	NR	14.5	NR	3.4	NR	2.6	NR	NR	92
Portugal (40)	112	25.9	1.8	9.8	2.7	9.8	1.8	25.9	4.4	6.3	1.8	0.3	88.4
USA (26)	364	31.9	0.8	23.4	NR	12.6	1.1	12.6	0.3	3.3	3.6	0.5	89.6
Argentina (27)	866	11.2	0.7	20.6	0.8	8.2	2.0	26.2	NR	6.7	4.2	1.4	82
Argentina (102)	1203	6.1	0.9	12.2	NR	8.6	0.7	42.6	0.4	4.4	2.2	2.8	94
Germany (47)	310	27.4	2.6	30.3	1.2	19.7	2.1	2.9	0.3	4.8	4.3	0.7	98.1
USA (28)	3005	20.0	3.7	22.9	2.1	8.2	0.3	23.9	1.1	3.5	7.4	NR	88.9
Brazil (22)	856	9.0	0.6	21.1	1.8	7.5	1.2	26.6	2.2	6.1	5.4	1.4	82.9
China (29)	310	27	1.9	29	1.0	12.9	1.9	4.2	2.3	4.8	5.5	NR	90.6

*Alleles (n): Number of alleles analyzed **Exon 6 cluster = p.[Ile237Asn; Val238Glu; Met240 NR: not reported.

Legacy names: p.(Pro31Leu) – P31L; c.293-13C>G – I2 splice; p.(Ile173Asn) – I172N; p.(Val282Leu) – V281L; p.(Gln319Ter) – Q318X; p.(Arg357Trp) – R356W; p.(Pro454Ser) – P454S.

Population-based genotyping studies have substantially advanced the molecular understanding of 21-OHD by delineating allele frequencies and genotype-phenotype correlations across diverse populations. To date, over 300 pathogenic variants have been identified in *CYP21A2*, including point pathogenic variants, small insertions/deletions, and large structural rearrangements (23). Across global cohorts, the c.293-13C>G (I2 splice) variant consistently emerges as the most prevalent pathogenic allele, accounting for approximately 20–25% of reported genotypes (24). This is followed in frequency by p.(Ile173Asn) (15–20%), p.(Val282Leu) (10-15%**),** p.(Gln319Ter) (5-10%), and p.(Pro31Leu) (3-5%), although notable regional variation persists (22, 25–29) (**Table 2**). Despite substantial geographic variability in individual pathogenic variants frequencies, pseudogene-derives variants consistently account for most disease-causing alleles across populations. In European and North American population, where long-standing newborn screening programs facilitate early detection, large gene deletions and *CYP21A1P*/*CYP21A2* chimeric conversions, predominantly associated with SW phenotypes, constitute 20-30% of pathogenic alleles (25, 28, 30, 31). Conversely, studies from Latin American countries such as Brazil, Mexico, and Argentina, which implemented systematic newborn screening only more recently, report lower frequencies (~8-12%) of large rearrangements (22, 27, 32). This discrepancy is likely attributable to historical underdiagnosis of severe cases in the pre-screening era and the resulting differences in the clinically ascertained cohorts available for genotyping.

Nevertheless, population-based studies continue to reveal rare or population-specific alleles, highlighting the extensive allelic heterogeneity of 21-OHD and the necessity of using ethnically tailored genetic panels for accurate diagnosis and effective genetic counseling (5, 16, 19, 22, 28, 33–35). The novel variants described in multiple studies encompass multiple alteration types including missense variants, p.(Gly424Ser), p.(Gly291Arg), p.(Ser301Tyr), p.(Arg483Gln), p.(Arg342Trp), nonsense variants, p.(Tyr376Ter), p.(Arg445Ter), frameshift variants (c.995-996insA, c.1123delC, c.1367delA), splicing variants and large deletions/conversions (26, 36–39). Functional characterization indicates most novel variants affect protein function through disruption of conserved residues or structural stability. Tardy et al. demonstrated that a series of rare missense substitutions variably impair 21-hydroxylase activity, ranging from near-null to residual levels compatible with nonclassical CAH, by disrupting heme coordination, active-site geometry, or overall protein stability (37). In a large Brazilian cohort, nine novel or rare variants were identified, several of which (p.(Arg408Cys), c.1450_1451insC, p.(His365Tyr), c.293-2C>G, p.(Gly424Ser) exhibited clear founder effects and shared haplotypes with Spanish and Portuguese patients, reflecting the historical Iberian contribution to Brazilian ancestry (22, 27, 40, 41). Founder effects have significantly influenced the global distribution of *CYP21A2* variants, shaping the mutational landscape of 21-OHD. In certain populations, specific pathogenic alleles became disproportionately frequent due to their origin in a small ancestral group, followed by genetic drift, geographic isolation, or population expansion. The p.(Val282Leu) variant, one of the most common causes of nonclassical CAH, illustrates this phenomenon, showing high prevalence in Mediterranean and Latin American populations due to Iberian migration, with a strong association with the Human Leukocyte Antigen (HLA) B14 allele (42, 43). The p.(Arg426His) variant demonstrates a similar founder pattern, described in Austrian and Spanish cohorts, where it consistently correlates with the salt-wasting phenotype and severe enzymatic impairment (41, 44). More recently, the p.(Gly424Ser) variant has been identified as a likely founder allele in certain European and South American cohorts (36, 41, 45), exhibiting recurrent haplotypes, strong linkage disequilibrium, and association with both simple virilizing and nonclassical phenotypes. Reports from Spanish and Chinese cohorts have added additional novel alleles supported by shared ancestral haplotypes and structural predictions of impaired protein stability (29, 34). These founder variants not only reflect the evolutionary and demographic history of affected populations but also carry significant implications for genetic counseling, carrier screening, and the interpretation of genotype-phenotype correlations in 21-OHD.

## Genotype-phenotype correlation

Many studies have investigated genotype-phenotype correlations in large national and multiethnic cohorts. In 21-OHD, a strong correlation between genotype and phenotype has been consistently reported across multiple populations (22, 26, 46, 47). Functional *in vitro* studies have allowed classification of *CYP21A2* pathogenic variants into four groups based on residual enzymatic activity: group 0 (null, 0%), group A (<2%), group B (3-7%), and group C (20-60%), as summarized in **Table 1** (18, 31, 48–50). This functional classification provides a useful framework for associating molecular defects with clinical presentation. Functional classification of *CYP21A2* variants is based on enzymatic activity measured *in vitro*; however, these estimates are derived from heterogeneous experimental systems, including different expression models (e.g., COS-1 or HEK293 cells), assay substrates, and normalization strategies. Consequently, reported activity values may vary across studies, and direct quantitative comparisons between variants should be interpreted cautiously, as they represent approximate functional ranges rather than strictly comparable measurements. In general, severe genotypes leading to SW uniformly showed a strong correlation with clinical phenotypes. Reported concordance rates range from 97-100% in null genotypes and 79-96% in A genotypes associated with the SW form, 53-87% in B genotypes associated with the SV form, and 65-100% in C genotypes with the NC form (19, 26, 31, 35, 47).

Most affected individuals are compound heterozygotes, and the clinical outcome is typically determined by the allele that maintains the highest residual enzyme activity. Consequently, the SW form generally results from combinations of two severe alleles (groups 0 or A), whereas the SV form arises in patients homozygous for group B variants, or compound heterozygous of group B variants with groups null or A. The NC phenotype usually manifests in carriers of two mild alleles (group C) or combinations of mild and severe (groups null, A or B) variants (22, 26, 47, 48).

However, the phenotypic spectrum is continuous, and boundaries between SW, SV, and NC forms are not always clear-cut, especially in males or in individuals with intermediate enzyme activity. Although genotype-phenotype correlation in 21-OHD is generally strong, discordant cases are well documented (25, 28) (see topic Pitfalls). Importantly, genotype-phenotype discordance in CAH reflects not only the primary *CYP21A2* pathogenic variant but also allelic configuration (cis/trans), promoter strength, splicing efficiency, copy number variation, and extra-adrenal metabolism. Therefore, apparent inconsistencies between genotype and clinical severity frequently result from incomplete molecular characterization rather than a true biological discrepancy.

## Molecular diagnostic methods

To date, a variety of molecular techniques have been used for the genetic analysis of 21-OHD. Overall, these techniques can be classified into three categories, which can be complementary (51):

- Analysis of duplications and deletions (Southern blot and MLPA).
- Targeted Genotyping Methods for Common *CYP21A2* Variants (Allelic specific dot blot, allelic specific Polymerase Chain Reaction (PCR), SnapShot minisequencing).
- Whole gene sequencing (Sanger sequencing, Next generation sequencing and Long-read sequencing).

### Analysis of duplications and deletions

Initial methodologies for molecular 21-OHD diagnosis relied on Southern blot. This technique enabled the detection of common *CYP21A2* deletions or conversions but lacked resolution for single-nucleotide variants. Subsequently, MLPA became a standard method for detecting deletions and large gene conversions.

### Southern blot

Southern blot represented a landmark technique in the molecular analysis of *CYP21A2*. By digesting genomic DNA and hybridizing specific probes, the method separates the two loci into distinct fragments, permitting identification of structural defects typically associated with severe, salt-wasting phenotypes (48). Despite its diagnostic value, Southern blot is labor-intensive and time-consuming, typically requiring 5–10 days depending on laboratory infrastructure, probe characteristics, and autoradiography exposure times. Early implementations relied on radioactively labeled DNA probes, raising significant concerns regarding laboratory safety and the long-term storage and disposal of radioactive phosphorus waste with prolonged half-lives. Subsequent developments introduced fluorescently labeled probes, allowing signal detection by fluorescence imaging systems with the advantages of digital quantification, improved safety, and multiplexing capabilities (52).

### Multiplex ligation-dependent probe amplification in *CYP21A2* genotyping

Introduced in the early 2000s, MLPA represented a major advance in the molecular diagnosis of CAH providing a rapid, sensitive, and non-radioactive method to detect copy-number variation (CNV) within the *CYP21A2* locus. MLPA replaces Southern blot by allowing simultaneous quantification of up to 50 genomic targets through multiplex probe hybridization, ligation, and fluorescent PCR amplification. Fragment analysis by capillary electrophoresis enables detection of heterozygous deletions, duplications, and *CYP21A1P*/*CYP21A2* chimeric alleles arising from unequal crossover. When combined with Sanger sequencing, MLPA provides near-complete genotypic resolution and remains the diagnostic gold standard recommended by European Molecular Genetics Quality Network (EMQN) guidelines (53). Because approximately 2-6% of affected alleles harbor two or more point variants, the combined MLPA-Sanger workflow requires parental segregation analysis to determine whether the variants are in cis or trans, a critical step for confirming compound heterozygosity and ensuring accurate genetic counseling (22, 25, 31, 54, 55). Although MLPA is highly reliable for dosage analysis, it cannot define breakpoints or resolve complex hybrid alleles at nucleotide resolution, being unable to distinguish attenuated chimeras CH-4 and CH-9 from classic chimera CH-6 (21, 56, 57), underscoring the emerging role of long-read sequencing technologies, which can directly phase variants and comprehensively characterize the *RCCX* locus in a single assay (58).

### Targeted genotyping methods for common CYP21A2 variants

Targeted Genotyping Methods for common *CYP21A2* variants relies on the fact that approximately 75% of pathogenic variants, especially SNVs and small insertions and deletions (Indels), are derived from nonfunctional pseudogenes by gene conversion events (56). These variants can be detected by specific molecular techniques because they either create or destroy specific DNA sequences or restriction sites. Several different methods and strategies have been described that cover a variable range of pathogenic variants.

The targeted genotype method was first described for 21-OHD by Speiser et al. in 1992 (48), utilizing allele-specific dot-blot hybridization with radioactive oligonucleotide probes, which was a labor-intensive and time-consuming approach. Allele-specific PCR (AS-PCR), the most widely adopted molecular assay for targeted detection of the frequent *CYP21A2* variants, was later described by Wedell and Luthman in 1993 (59). In 1994, Speiser et al. extended allele-specific hybridization to prenatal diagnosis, providing one of the first clinically validated targeted genotyping strategies for recurrent *CYP21A2* variants (60). This work established its translational utility and paved the way for subsequent allele-specific PCR, SNaPshot minisequencing, and other targeted pathogenic variant panels used in routine diagnostic workflows.

**Figure 2:**
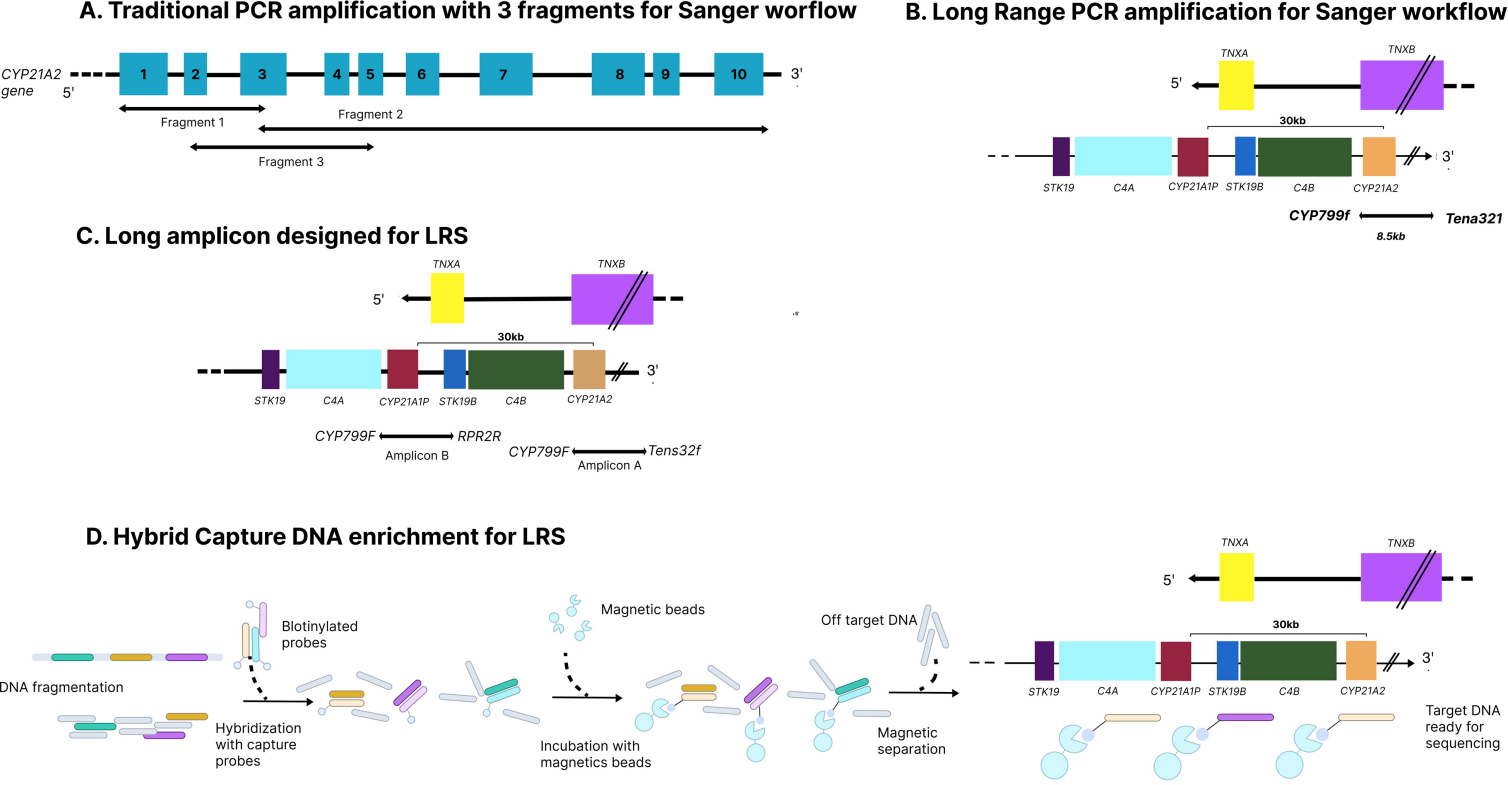
Methodologies of DNA amplification or enrichment prior to CYP21A2 sequencing. **(A)** Traditional PCR-based amplification using three overlapping fragments covering the CYP21A2 gene, followed by Sanger sequencing; this approach relies on exon-specific primers and is vulnerable to allele dropout and pseudogene interference. **(B)** Long-range PCR strategy for Sanger sequencing, in which a single large amplicon spans the CYP21A2 gene and adjacent RCCX elements, extending toward the TNXB region to improve discrimination from the CYP21A1P pseudogene. **(C)** Long-amplicon design for long-read sequencing (LRS), enabling continuous sequencing across CYP21A2, CYP21A1P, C4 genes, and RCCX junctions, thereby allowing accurate detection of single-nucleotide variants, indels, and structural rearrangements. **(D)** Hybrid capture–based DNA enrichment for PCR-free LRS, in which fragmented genomic DNA is selectively captured by biotinylated probes and magnetic beads, allowing comprehensive analysis of the RCCX locus, including copy-number variations, gene conversions, and complex chimeric alleles. **(D)** Long-read sequencing enables direct haplotype phasing, allowing unambiguous determination of cis/trans configuration of CYP21A2 variants without the need for parental testing. This schematic representation is not drawn to scale.

The first challenge in detecting variants within *CYP21A2* is ensuring the specific amplification of the functional gene rather than its highly homologous pseudogene (*CYP21A1P*) (61). This specificity relies on designing primers that target regions uniquely divergent from the pseudogene, most commonly within exon 3, where the *CYP21A1P* pseudogene carries the characteristic variant c.332_339del (8 bp deletion). Therefore, a commonly adopted strategy for gene-specific amplification of *CYP21A2* involves generating two fragments: one extending from the 5′UTR to exon 3, and another from exon 3 to the 3′UTR. However, this approach may fail to detect alleles harboring the c.332_339del variant as an isolated microconversion, as well as chimeric genes, conversion events, or duplications that include this region. To overcome this limitation, a third overlapping fragment can be incorporated, in which the 3′ primer is located within exon 6, a region containing a cluster of three characteristic variants in *CYP21A1P*. The inclusion of this additional fragment enables the PCR-based detection of pseudogene-gene chimeras with breakpoints occurring upstream of exon 6 (11, 61) (**Figure 2**).

After the gene amplification, the second PCR is allele specific; for each pathogenic variant of interest, two parallel reactions are carried out using primers designed for the normal and mutant alleles, respectively. A third internal control primer pair is frequently added to amplify a larger product, confirming the quality of each reaction (62).

An evolution of targeted genotyping methods is the multiplex snapshot minisequencing assay, enabling multiplex detection of several known *CYP21A2* point variants in a single reaction. Following PCR amplification, primers anneal adjacent to each variant site and incorporate a single fluorescent labeled dideoxynucleotide (ddNTP), which is subsequently resolved by capillary electrophoresis to identify the corresponding nucleotide. This single-base extension strategy offers higher throughput and multiplexing capacity than traditional AS-PCR. The method shows remarkable precision, with Krone et al. reporting 100% concordance in detecting common variants. The technique was notably rapid, proved by the ability to analyze 40 patient DNA samples in just 1.5 working days (63). Keen-Kim et al. further validated this strategy by employing a locus-specific PCR using four primers located outside the open reading frame to assess the presence of genomic rearrangements or deletions. This was subsequently followed by minisequencing with internal primers to identify pathogenic variants in *CYP21A2*. The method was evaluated in 20 previously genotyped samples and 19 control samples, confirming its reliability for clinical diagnosis and prenatal testing (64). Tokarska et al. additionally noted the method’s superiority in being faster and more comprehensive compared to traditional single SNP detection methods (65).

Other targeted methods used for detecting recurrent *CYP21A2* variants include Allele-Specific Oligonucleotide (ASO) hybridization, Restriction Fragment Length Polymorphism (RFLP) assays, Denaturing High Pressure Liquid Chromatography, Single-Stranded Conformation Polymorphisms and Amplification-Created Restriction Sites. These techniques share the principle of discriminating known variants through hybridization or enzyme-based recognition rather than sequencing (27, 51, 66–69). ASO hybridization uses short, labeled DNA probes specific to either the normal or mutant sequence and provides high specificity and suitability for high-throughput screening; however, it requires stringent optimization of hybridization conditions and careful probe design to avoid pseudogene cross-reactivity (70). RFLP analysis, among the earliest molecular tools for *CYP21A2* testing, is inexpensive and technically simple, yet it detects only variants that create or abolish restriction sites and offers low resolution for complex alleles (54).

In most populations, these methods achieve a detection rate of approximately 90-95% of known pathogenic alleles, providing a balance between analytical precision, efficiency and reproducibility, making it suitable for detecting recurrent *CYP21A2* variants in diagnostic laboratories during the 1990s and early 2000s (27, 31, 47, 71, 72). They are suitable for carrier testing, confirmation of newborn screening results, and rapid genotyping in populations with well-characterized founder variants. Despite its robustness, they are limited to predefined variants and cannot identify novel or complex alleles, large deletions, or gene conversions that are frequent within the *RCCX* locus (73). Recently, the specific allele-targeted method has been largely replaced by comprehensive genotyping approaches such as Sanger sequencing. Nevertheless, it remains a valuable historical milestone in the evolution of molecular diagnostics for CAH and continues to be used in some targeted pathogenic variant screening panels.

### Whole gene sequencing

The emergence of direct sequencing technologies, allowing complete examination of the *CYP21A2* coding region and its intronic boundaries, marked a crucial transition from variant-targeted assays toward comprehensive gene-level analysis, thereby setting the foundation for the modern era of molecular diagnosis in CAH.

### Sanger sequencing in the molecular diagnosis of *CYP21A2*

Sanger sequencing marked a breakthrough in CAH molecular diagnosis by enabling single-base resolution analysis of *CYP21A2*. It allowed comprehensive detection of common and rare pathogenic variants, including point variants, indels, and microconversion-derived changes, that were inaccessible to earlier allele-specific or hybridization methods.

This method is also preceded by the direct amplification of *CYP21A2* by locus-specific long-range PCR protocols, employing primers located in divergent regions between the gene and pseudogene. Two or three overlapping amplicons typically cover the full coding sequence and intron-exon junctions of *CYP21A2*, providing complete and specific template material for sequencing reactions. This design ensures clear separation from pseudogene artifacts and yields highly reproducible chromatograms for base calling and zygosity determination (74). A well-established protocol for sequencing the *CYP21A2* gene is the use of long-range PCR that amplifies an 8.5kb fragment (the *CYP779f/Tena32F* amplicon) encompassing the full *CYP21A2* gene and part of the *TNXB* gene. Compared with the targeted common variant analysis approaches, whole gene (coding regions and canonical splicing sites) sequencing can reveal both common and rare variants. This 8.5kb *CYP779f/Tena32F* amplicon can reveal *TNXA*/*TNXB* chimeras and has been utilized to study patients with the CAH-X syndrome, characterized by features of both 21-OHD CAH and connective tissue issues, specially the hypermobility Ehlers-Danlos Syndrome (75).

For more than three decades, Sanger sequencing has been regarded as the gold standard for *CYP21A2* analysis and remains the reference method in diagnostic and research laboratories worldwide (22, 47, 53). Historically, it provided the basis for the development of *CYP21A2* variant databases, genotype–phenotype correlations, and the molecular classification of CAH subtypes. When combined with complementary methods such MLPA for copy number analysis, it provides nearly complete diagnostic coverage, identifying up to 98-99% of classical pathogenic alleles in most populations (1, 16, 76). It also provides superior base-level accuracy, excellent reproducibility across laboratories, and robust traceability of results.

For a comprehensive molecular characterization, sequencing of both exonic and intronic regions, as well as the proximal promoter, is essential. Pathogenic variants in *CYP21A2* are not confined to the coding exons; several intronic splice-site variants (such as the canonical intron 2 variant c.293-13C>G), as well as promoter-region substitutions (e.g., c.-113G>A and c.-126C>T), have been shown to significantly interfere with gene expression or splicing efficiency (77). Extending Sanger sequencing beyond the exons allows accurate detection of these regulatory and noncoding variants, many of which influence residual enzyme activity and modify the clinical phenotype, being responsible for some genotype-phenotype discordance. The identification of junction sites of chimera genes also can also explain some genotype-phenotype discrepancies. Sequencing the well-established *CYP779f/Tena32F* amplicons is an unequivocal strategy for detecting attenuated chimeric *CYP21A1P*/*CYP21A2* genes, which are clinically relevant (78, 79). Consequently, full *CYP21A2* sequencing, including exons, intron-exon boundaries, and promoter regions, provides a more accurate genotype-phenotype correlation and prevents underestimation of mild or compound alleles.

Its per-sample cost is higher than multiplexed NGS panels when large cohorts are analyzed, but it is a cost-effective method for single-gene disorders such as 21-OHD, particularly in laboratories already equipped for traditional sequencing methods. The method requires relatively inexpensive reagents and standard capillary electrophoresis instruments. Nevertheless, the method can be labor-intensive and time-consuming, requiring separate amplification reactions for each gene fragment and remains with some limitations. Sanger sequencing lacks allelic phasing, frequently requiring parental testing, and is also susceptible to allele dropout, a PCR-related limitation that should be considered when heterozygous variants are not detected. Also, the long-range PCR amplicon used to sequence CYP21A2 alone cannot reliably detect duplication alleles; therefore, an additional locus-specific amplicon may be required. In routine diagnostic workflows, this limitation is often addressed by combining MLPA with Sanger sequencing, which enables detection of copy number changes, including duplications (11, 21).

In the specific case of *CYP21A2*, Sanger sequencing often outperforms short-read NGS despite the latter’s higher throughput. Short-read NGS struggles with misalignment due to the near-identical sequence of *CYP21A1P*, leading to false variant calls or allele drop-outs unless gene-specific enrichment and specialized bioinformatic filters are applied.

In contrast, Sanger sequencing directly analyses PCR-amplified *CYP21A2* fragments, completely avoiding pseudogene interference and ensuring unambiguous variant confirmation. Its superior per-base accuracy and established performance make it the benchmark for clinical validation of any novel variant detected by NGS (80). Even in the era of high-throughput sequencing, Sanger-based workflows continue to be recommended by international guidelines such as the EMQN for confirmatory testing and variant verification (53).

### Next-generation sequencing in *CYP21A2* genotyping

The introduction of NGS technologies seemed promising for molecular diagnostics since it enables simultaneous analysis of multiple genes with high throughput and reduced cost per base (81, 82). The NGS assay has increased the diagnosis efficacy for diseases associated with multiple genes (83). These characteristics are of interest in newborn screening and carrier status screening for prenatal advice (84, 85). In CAH, targeted panels or whole-exome sequencing (WES) can identify variants in *CYP21A2* and other steroidogenic genes (*CYP11B1, HSD3B2, CYP17A1, POR, STAR*), improving differential diagnosis between 21-OHD and other rare CAH subtypes with overlapping phenotypes and enabling variant discovery (86). However, the *CYP21A2* locus remains technically challenging for short-read NGS platforms due to its high sequence homology with the pseudogene *CYP21A1P*, leading to frequent mapping errors and potential allele misclassification (85). Most NGS workflows are based on short-read sequencing technologies, which, in the *CYP21A2* gene, often align ambiguously between the functional gene and the pseudogene because of their known high sequence similarity. As a result, reads can be erroneously assigned, producing both false-positive and false-negative variant calls. Moreover, short-read sequencing lacks the ability to identify large gene conversions, duplications, and chimeric *CYP21A1P/CYP21A2* alleles, which account for a significant proportion of pathogenic variants (85). To overcome these limitations, a combination of laboratory and bioinformatics strategies is commonly employed to improve CYP21A2-specific resolution, including pseudogene masking during data analysis, locus-specific long-range PCR for selective amplification, and targeted hybrid-capture panels prior to library preparation (81, 82, 85). These strategies selectively enrich the functional *CYP21A2* gene, preventing pseudogene interference and enabling accurate variant calling. In targeted or exome-based NGS panels, the diagnostic yield for *CYP21A2* can approach that of Sanger sequencing when complemented by copy-number detection methods such as MLPA. Few studies found significant advantages using NGS for CAH diagnosis: Wang et al. achieved 98.5% successful genotyping across nine CAH candidate genes, identifying 125 variants including missense (46.8%), splicing (21.5%), and structural variations (11.8%) (82) and so Gangodkar et al. showed 82.6% detection of biallelic variants in 310 CAH cases, with 100% confirmation by capillary sequencing or MLPA (80). When properly optimized, short-read NGS provides high diagnostic accuracy and scalability. In *CYP21A2* genotyping, however, performance depends on the sequencing design. Multigene panel approaches may be affected by pseudogene interference, whereas *CYP21A2*-targeted strategies using locus-specific enrichment (e.g., long-range PCR or targeted capture) have shown reliable detection of pathogenic variants. In this context, Sanger sequencing is mainly used for confirmation of selected findings or in cases of diagnostic uncertainty.

### Long-read sequencing and future perspectives

LRS has taken a central role in recent years, enabling comprehensive analysis of genomic regions that are difficult to resolve with traditional methods. This third-generation sequencing can achieve read lengths ranging from 15 kb to 2 Mb for DNA sequencing, representing orders-of-magnitude longer read lengths compared with short-read NGS platforms (75). By generating contiguous kilobase-scale reads, LRS allows precise characterization of genome rearrangements, repetitive sequences, and regions of high sequence identity, such as the *CYP21A2* locus and its highly homologous pseudogene *CYP21A1P* (84, 87). These capabilities permit unambiguous gene-pseudogene discrimination, accurate breakpoint mapping, and direct haplotyping (cis/trans determination) within a single assay, thereby overcoming the main limitations of both short-read NGS and classical two-step workflows (Sanger + MLPA).

In the context of CAH, LRS has demonstrated superior diagnostic performance compared with traditional methods. Liu et al. demonstrated that a comprehensive CAH molecular workflow with LRS reached 100% sensitivity and specificity, fully matching the diagnostic performance of the conventional MLPA plus Sanger sequencing approach (75). Wang et al. confirmed these findings in a large cohort of 322 probands, with LRS improving precise genotype assignment in 14.6% of cases, by accurately classifying six common deletions (*CYP21 CH-3*, *CH-5*, *CH-8* and *TNX-CH1*, *CH2*, *CH3*) that are challenging to detect using MLPA or Sanger sequencing (88). Similarly, Yuan et al. showed that LRS can directly identify pathogenic variants and resolve complex chimeric configurations that traditional techniques cannot, underscoring its clinical value in *CYP21A2* genetic characterization (89).

In a recent study by Claps et al., LRS was used in an Argentinian cohort to dissect the complex *RCCX* locus harboring the *CYP21A2* gene. The workflow identified not only all the expected pathogenic variants previously described by standard methods (Sanger/MLPA) but also novel variants not previously described in Latin American populations (19 in the pseudogene *CYP21A1P* and 29 in the *TNXA*) and reclassified chimeric or converted alleles in 18.75% of samples through precise breakpoint mapping. This work underscores LRS’s utility not only in diagnosing known pathogenic alleles but also in expanding the mutational spectrum of *CYP21A2* and improving our understanding of *RCCX* structural diversity in a population-specific context, with direct implications for carrier screening, genotype–phenotype correlations and tailored genetic counselling (90).

Technically, long reads begin and end within *CYP21A2*-specific sequences, eliminating the misalignment artifacts common to short reads. Unlike short-read NGS, whose 100–300 bp reads frequently mis-map between *CYP21A2* and the highly homologous pseudogene *CYP21A1P*, LRS generates long kilobase-scale reads (often exceeding 10 kb) with high per-read accuracy (with PacBio HiFi reads providing accuracies comparable to or exceeding Q30), allowing unambiguous gene-pseudogene discrimination. They span large rearrangements, breakpoints, and *CYP21A1P/CYP21A2* chimeric junctions, defining their exact structures without inference. Variant phase (cis/trans) is observed directly on single molecules, reducing or eliminating the need for parental segregation testing in most cases.

Current clinical workflows typically combine long-range PCR or targeted hybrid capture for enrichment of the *CYP21A2*/*RCCX* region, followed by sequencing on PacBio HiFi or Oxford Nanopore platforms. These assays can simultaneously detect all variant classes (SNVs, indels, CNVs, gene conversions, and hybrid alleles), while providing haplotype-level data that streamlines interpretation (12, 90). Beyond PCR-based enrichment, hybrid-capture approaches have recently been combined with long-read platforms to selectively isolate the *CYP21A2* locus and adjacent *RCCX* components prior to sequencing. By enriching target molecules and increasing allelic representation, hybrid capture markedly enhances coverage uniformity and enables more precise discrimination between *CYP21A2* and its highly homologous pseudogene (**Figure 2**). In the comparative analysis by Lan et al., hybrid capture combined with long-read sequencing identified complex rearrangements, chimeric alleles, and phasing configurations that were frequently missed by long-PCR and MLPA workflows, underscoring the superiority of LRS-based strategies for comprehensive *CYP21A2* characterization (75).

Despite its advantages in read length and structural resolution, long-read sequencing (LRS) still faces limitations for routine diagnostics, particularly for PacBio-based platforms, which require high-quality DNA, higher costs, and substantial computational resources for Circular Consensus Sequencing (CCS). In contrast, Oxford Nanopore Technologies (ONT) provides more cost-effective and portable sequencing solutions and has already been successfully applied to *CYP21A2* genotyping, demonstrating its feasibility for complex loci analysis. Remaining challenges mainly relate to data analysis and standardization rather than technical applicability (90). These factors continue to restrict widespread implementation in routine diagnostics (75). Although current costs remain higher than traditional essays, batching and targeted panels are rapidly improving affordability. By consolidating multiple analytical steps into a single comprehensive assay, LRS streamlines workflow and shortens turnaround time, highlighting its potential applicability to future CAH newborn screening strategies. Its integration could provide complete genotyping in a single test, reducing delays and substantially lowering false-positive rates (87).

As throughput increases and costs continue to decline, LRS may emerge as a first-tier diagnostic tool for CAH, particularly in centers managing heterogeneous or complex genotypes. In the meantime, hybrid diagnostic workflows remain the practical clinical standard, generally combining MLPA and Sanger sequencing for first-line testing, followed by targeted NGS or LRS for unresolved or ambiguous cases. Growing evidence indicates that LRS may progressively assume a central role in CAH diagnostics, particularly in complex or unresolved cases, although current clinical practice still relies on Sanger plus MLPA in most settings.

A comparative overview of the analytical capabilities, advantages, and limitations of Sanger sequencing, MLPA, short-read NGS and long-read sequencing is summarized in **Table 3**.

**Table 3:** Comparative performance of molecular methods for CYP21A2 analysis.

Molecular diagnostic techniques	Detects SNV	Detects CNV	Detects chimeras	Phasing (cis/trans)	Limitations
Sanger sequencing	✓	×	×	×	Cannot detect large rearrangements
MLPA	×	✓	Partial (indirect)	×	Limited breakpoint resolution
Short-read NGS	✓	partial	limited	×	Misalignment with pseudogene
Long-PCR + Sanger*	✓	partial	Partial (via targeted amplification)	×	Requires locus-specific long-range amplification; may still miss complex rearrangements
Hybrid capture + short-read NGS	✓	✓	improved	×	Still mapping constraints
Long-read sequencing	✓	✓	✓	✓	Higher cost and higher DNA quality requirement

The terms “partial”, “limited” and “improved” indicate relative detection sensitivity and resolution compared with long-read sequencing, which represents the reference method for comprehensive CYP21A2 variant characterization. * Long-range PCR + Sanger differs from standard locus-specific Sanger sequencing in the amplification strategy. While conventional Sanger sequencing typically uses two or three overlapping locus-specific PCR amplicons covering the CYP21A2 gene, long-range PCR uses a 8.5-kb fragment using CYP779f/Tena32F primer that encompass the entire CYP21A2 gene and part of TNXB.

## Pitfalls in CAH-21OHD molecular diagnosis

Molecular diagnosis of 21-OHD presents unique challenges due to the exceptional genomic complexity of the *CYP21A2* locus. Diagnostic pitfalls arise at multiple levels, from assay design and amplification bias to the biological consequences of allelic configuration, regulatory variants, and secondary compensatory pathways.

### Mechanisms of genotype-phenotype variability

Genotype-phenotype variability in 21-OHD reflects both the primary defect and locus-level architecture of the *RCCX* module (1). The intron-2 splice variant (c.293-13C>G, “I2 splice”), one of the most common variants among different populations, reduces correct splicing by strengthening a cryptic acceptor and promoting aberrant exon-3 inclusion/skipping, normally leading to severe disease; however, the clinical presentation can vary from the SW form, through the SV form and rarely the NC form. The most accepted hypothesis for that is a small number of transcripts avoiding aberrant splicing, providing a small amount of the wild 21-hydroxylase enzyme, which is sufficient for a milder clinical presentation of the disease (24). The variant p.(Gln319Ter), historically referred to as Q318X, typically results in complete loss of enzymatic activity. However, it may be present within a duplicated *CYP21A2* loci, containing both a wild-type *CYP21A2* copy and a second *CYP21A2* copy carrying the pathogenic variant. In such cases, enzymatic activity of the 21-hydroxylase is preserved, thus representing a normal functional allele. This structural configuration may lead to apparent genotype misclassification if copy number analysis is not performed (91). Promoter alterations, including microconversions or point substitutions in upstream regulatory regions (such as c.-126C>T, c.-113G>A, c.-110T>C, and c.-103A>G), reduce transcriptional output and can modify the phenotypic impact of missense variants like p.(Pro31Leu), shifting expected NC/SV presentations toward increased androgen excess as promoter activity declines (78). In addition to promoter variants, pathogenic variants have also been described in the 3′ untranslated region (3′UTR), including c.*13G>A, which may affect gene expression and clinical presentation, being associated with a mild CAH phenotype (92). In attenuated chimeras whose junctions lie upstream of c.293-13C>G, the recombinant allele inherits the pseudogene promoter plus c.89C>T (p.(Pro31Leu)), often producing NC or SV even though a large conversion is present (28). The variant p.(Val282Leu), typically associated with the nonclassical form, has been reported in salt-wasting cases when present in cis with a rare intronic splice variant (c.292 + 5G>A) described in Mediterranean cohorts. In this context, the increased severity is likely driven primarily by the splicing defect, which further reduces residual enzymatic activity below the threshold associated with the nonclassical phenotype, rather than by p.(Val282Leu) alone (40, 93). The variant p.(Ile173Asn) shows interindividual variability likely driven by subtle differences in mRNA abundance, translation efficiency, and co-segregating regulatory variants affecting enzyme dosage (22, 28). Recognizing and addressing these discordances is essential for accurate clinical interpretation, individualized management, and appropriate genetic counselling in CAH (71, 72, 94).

Finally, extra-adrenal 21-hydroxylation via liver P450 cytochromes (*CYP2C19/CYP3A4*) can partially compensate in some contexts, blunting biochemical severity and contributing to clinical discordance (95). Additionally, modifier genes, including androgen receptor polymorphisms and variations in cytochrome P450 oxidoreductase (POR, the electron donor for *CYP21A2*), can influence steroid metabolism and clinical expressivity. All these mechanisms help explain divergent phenotypes in patients with the same genotype, emphasizing the need for cautious and integrated interpretation of molecular findings (16).

Taken together, these mechanisms illustrate why LRS, by resolving full allele structure, promoter status, junction breakpoints, and cis/trans phase within a single assay, may markedly reduce misclassification and improve concordance between molecular findings and clinical or biochemical data. Nevertheless, accurate diagnosis still requires expert interpretation and integration with the patient’s phenotype (11).

### Structural and interpretative pitfalls

Beyond the effect of individual variants, *CYP21A2* genotyping may be complicated by large deletions and gene conversions that generate *TNXA/TNXB or CYP21A1P/CYP21A2* chimeric alleles through non-allelic homologous recombination. *TNXA/TNXB* chimeras eliminate the entire *CYP21A2* gene and part of *TNXB*, whereas *CYP21A1P/CYP21A2* chimeras transfer pseudogene sequences into the coding region, frequently introducing severe splice or coding defects. To date, eleven *CYP21A1P/CYP21A2* chimeras have been defined based on breakpoint mapping within the *RCCX* module. Functionally, these chimeric alleles can be grouped into three clinically meaningful subcategories: attenuated CAH chimeras, which only moderately impair the affected allele; classic CAH chimeras, which nullify *CYP21A2;* and CAH-X chimeras, in which the rearrangement extends into *TNXB*, causing a contiguous syndrome of 21-OHD plus hypermobility Ehlers–Danlos syndrome (16, 79, 96, 97). Regarding the most frequently occurring chimeras (CH1-CH9), when the breakpoint lies downstream of the c.293-13C>G (I2 splice) variant, the resulting chimeras usually include this severe variant (*CH1, CH2, CH3, CH5, CH6, CH7, CH8*). Conversely, chimeras whose junction occurs upstream of the c.293-13C>G site (*CH4* and *CH9*) typically contain the *CYP21A1P* promoter and p.(Pro31Leu) and are associated with milder SV or NC phenotypes (21).

These structures can be overlooked or misinterpreted by traditional workflows, leading to apparent genotype–phenotype discordance. In addition, pseudogene interference, mis-amplification, and copy-number variation may yield spurious variants or mask deleterious alleles, while lack of phasing information can prevent discrimination between compound heterozygosity and complex cis alleles. Together, these factors underscore why structural characterization and accurate haplotyping are essential for reliable CAH molecular diagnosis (11, 51).

### Methodological limitations and modern solutions

Traditional workflows (Sanger/MLPA) accurately detect point variants and exon-level CNVs but cannot map promoter conversions, determine breakpoint architecture, or resolve cis/trans relationships. Short-read NGS increases throughput but shows limitations within the *RCCX* locus due to >98% sequence homology between the gene and its pseudogene. In addition to pseudogene interference, GC-rich regions within the *CYP21A2*/TNXB segment contribute to amplification and sequencing bias, frequently resulting in uneven coverage, allele drop-out, and misalignment when PCR-dependent or short-read approaches are used. This GC-bias can mask low-frequency alleles, produce apparent homozygosity, and hinder detection of complex conversions, further complicating molecular interpretation of *CYP21A2*. These limitations contribute to apparent genotype-phenotype discrepancies and underdiagnosis of complex alleles.

LRS now can overcome these barriers by spanning entire *CYP21A2-CYP21A1P* modules, detecting point variants, structural variants, gene conversions, and promoter alterations while providing direct haplotype phasing. Several studies show that LRS frequently reclassifies previously unresolved or misinterpreted cases, resolving many clinical-molecular mismatches (89). Hybrid-capture enrichment further improves coverage uniformity by reducing the impact of GC-bias and enhancing discrimination between gene and pseudogene, particularly in PCR-free long-read workflows (58).

Most genotype-phenotype discrepancies reflect incomplete molecular characterization rather than true biological discordance. Accurate diagnosis requires integrating sequencing results with knowledge of transcriptional effects, allelic configuration, and biochemical phenotype. By combining LRS-based genotyping with expert clinical interpretation, CAH diagnosis becomes more precise and aligned with individualized patient management.

## From molecular biology to clinical practice

Although 17-OHP measurement is the standard first-line newborn screening tool for 21-OHD, its interpretation is challenging in several real-world scenarios, including premature infants, individuals previously exposed to glucocorticoids, and patients whose biochemical features overlap between classic and nonclassical CAH (1).

Molecular analysis reduces false-positive newborn screening referrals, clarifies ambiguous hormonal profiles, and identifies individuals carrying severe loss-of-function alleles (<2% residual enzyme activity) who may benefit from early prophylactic management to prevent adrenal crises. Beyond diagnosis, genotyping enhances prognostic accuracy, informs therapeutic planning, and supports reproductive counseling, positioning CAH-21OHD as a model of precision endocrinology.

Molecular testing is being considered as a second or third tier in CAH newborn screening following immunoassay and/or LC-MS/MS (56). By resolving ambiguous biochemical results and distinguishing transient hyper-17-OHP from true disease, genotyping reduces false positives rates, unnecessary hospitalization, and parental anxiety (1, 16, 22, 81). When integrated with steroid profiling, molecular confirmation markedly enhances screening performance, improves early detection of salt-wasting cases, and enables prognostic stratification based on predicted residual enzyme activity. Population-based studies demonstrate that molecular integration enhances sensitivity, reduces false positives by up to 32%, and facilitates lowering 17-OHP cutoffs without compromising specificity, benefits unattainable with biochemical approaches alone (98).

Nonetheless, important operational challenges remain. Multiple *CYP21A2* variants in cis cannot be phased in newborn dried-blood spots, limiting discrimination between carriers and affected individuals and requiring confirmatory testing when ≥2 variants are detected. Lower 17-OHP thresholds increase sensitivity but impose larger molecular reflex workloads, with implications for cost, laboratory capacity, and turnaround time. These limitations underscore the need for scalable sequencing workflows and emerging long-read or phasing technologies, while hybrid biochemical-genetic models continue to reshape CAH newborn screening (98).

The combination of long-range PCR and long-read sequencing approaches near-complete sensitivity and specificity, with significant reductions in false positives and turnaround time, while maintaining competitive costs (<USD 20/sample) (75, 99). With ongoing automation and cost reduction, hybrid models integrating biochemical and genetic markers from the first screening tier are technically feasible in several settings. The integration of biochemical and genetic markers is redefining CAH newborn screening, merging diagnostic precision, individualized prognosis, and cost-effectiveness.

Also, molecular diagnosis has major implications for reproductive counselling and assisted reproduction. Identification of carrier status in parents enables accurate recurrence-risk estimation and informs decisions regarding family planning (1). Prenatal therapy for CAH is controversial because only affected female fetuses derive benefit, meaning that most at-risk pregnancies would be unnecessarily exposed to corticosteroids. Although molecular diagnosis can guide targeted treatment, conventional prenatal testing relies on invasive procedures such as chorionic villus sampling or amniocentesis, which carry procedural risks (16, 76). Noninvasive cell-free fetal DNA testing enables fetal sex determination and genotyping as early as 4–5 weeks of gestation, allowing prenatal therapy to be limited to affected females only (16, 100).

*In vitro* fertilization with preimplantation genetic testing permits selection of unaffected embryos in carrier couples, effectively preventing transmission of classic CAH and reducing the need for prenatal corticosteroid exposure. For couples with a child previously affected by salt-wasting CAH, early genotyping supports individualized reproductive counselling, optimizes prenatal management strategies, and expands reproductive options. As molecular methods become more widely available, integration of *CYP21A2* genotyping into genetic counselling protocols represents a critical step in modernizing CAH care and mitigating the intergenerational burden of disease (76, 101).

## Conclusion

Over the past decades, molecular diagnosis of congenital adrenal hyperplasia due to 21-OHD has evolved from labor-intensive, variant-focused methods to comprehensive genomic approaches capable of resolving the full complexity of the *CYP21A2* locus. This technological trajectory, from allele-specific assays and Sanger sequencing to MLPA and, more recently, long-read sequencing, has progressively overcome the challenges imposed by pseudogene interference and structural rearrangements, enabling accurate identification of all variant classes in a single analysis. Beyond technical refinement, these advances are reshaping clinical practice by improving diagnostic precision, guiding individualized management, enhancing newborn screening algorithms, and strengthening genetic counseling and reproductive planning. As long-read continues to mature and integrated with emerging bioinformatic tools, the field is moving progressively converging toward a unified, high-resolution diagnostic standard that supports the broader vision of precision endocrinology across the lifespan in congenital adrenal hyperplasia.
